# Equipoise and research in the current COVID-19 pandemic

**DOI:** 10.1017/cts.2020.48

**Published:** 2020-05-15

**Authors:** Jill M. Pulley, Rebecca N. Jerome, Todd W. Rice, Christopher J. Lindsell, Paul A. Harris, Terri L. Edwards, Consuelo H. Wilkins, Gordon R. Bernard

**Affiliations:** 1 Vanderbilt Institute for Clinical and Translational Research, Vanderbilt University Medical Center, Nashville, TN, USA; 2 Vanderbilt Center for Lung Research, Vanderbilt University Medical Center, Nashville, TN, USA; 3 Department of Biostatistics, Vanderbilt University, Nashville, TN, USA; 4 Department of Medicine, Vanderbilt University Medical Center and Department of Internal Medicine, Meharry Medical College, Nashville, TN, USA

**Keywords:** COVID-19, SARS-CoV-2, clinical trials, research infrastructure, scientific evidence

Equipoise in translational research and in clinical practice means there is essential uncertainty in terms of benefit or risk resulting from the use of a particular therapy. The interplay between equipoise and ethics forms one of the essential foundations of clinical research; one notable clinical ethicist succinctly characterized equipoise as “a clinically necessary condition in all cases of clinical research” [1]. Likewise, in the absence of systematic investigation such as through randomized controlled clinical trials (RCTs) and other rigorous methods, clinical actions are based on belief. RCTs have been the formal regulatory basis for new drug approvals for almost 60 years [2], and the gold standard since the first RCT was conducted in 1747 [3].

In the setting of COVID-19, there are no therapies with known effect. We rely on supportive care only. To be clear, 100% of pharmacological therapies in use today are not established for this disease – no evidence exists that any treatment is more helpful than harmful, a critical premise underlying the ethical practice of medicine. A range of unsubstantiated calls for use of interventions developed for management of other infectious diseases have propagated and led to significant confusion among providers and other stakeholders during this pandemic. Despite a multitude of observational/retrospective studies, pre-prints that have not yet been peer-reviewed, anecdotes, and reports on open-label use, we continue to have a murky picture of which approaches might be helpful for specific patients at different stages of the disease. This is further conflated by an emerging picture, albeit with similar limitations, showing potential harms associated with the use of interventions believed to be beneficial. The fear is that these therapies might cause harm and have no demonstrable benefit, and without rigorous testing, we have nothing to argue otherwise. Fundamentally, a firm evidence base on which to decide the appropriate path for the patients in our care is lacking, and the abundance of anecdote – normally a hypothesis *generation* mechanism – can get in the way of our societal need to understand the risks and benefits of untested therapies through well-controlled, RCTs.

The challenges facing us in the current pandemic are undeniably urgent, but do not preclude systematic and thorough evaluation of risk and benefit. This call is echoed by others advocating for design and completion of high-quality trials to evaluate the safety and efficacy of all therapies under consideration for COVID-19 [4–8]. In fact, the agility and collective strength of our national research infrastructure can enable rapid adaptation of our established methods and breadth of investigator expertise to quickly develop and implement RCTs to assess investigational approaches for COVID-19 disease. If academic medical centers have the ability to do so, new therapeutic ideas for management of COVID-19 should be investigated, rather than relying on off-label use. Of course, this is easier said than done, requiring a comprehensive and pragmatic application of research infrastructure to the unique challenges of the pandemic. The organization needed to conduct research in this setting includes a strong and supportive research infrastructure, expert protocol drafting sprints, dedicated and efficient Institutional Review Boards (IRB), swift evidence review, substantial recruitment support including for non-English speaking populations that might be at greatest risk, mechanisms for seeking and integrating ideas from clinical ethicists and other experts, regulatory and investigational new drug drafting experience, flexible and facile investigational drug pharmaceutical services, capabilities for rapid implementation in clinical information systems (such as for randomization schema and order sets), statistical design expertise, automated data capture tools, regulatory-acceptable eConsenting tools, skilled coordination staff, efficient contracts management, patient engagement, dexterity in dissemination of findings, and if multisite, single IRB structure and associated reliance agreements, as well as contractual agreements. Mechanisms for rapid funding from the federal government and other sources are critical. Finally, the importance of research literacy at every level of the healthcare system and by extension the communities we serve cannot be overlooked, both for the current pandemic and for the future of clinical research. The training and knowledge of frontline providers who are also researchers are paramount in moving ahead with a sound research agenda. There are key roles in informing these efforts for all those on the frontline – nurses, mental health professionals, case workers, pharmacists, operational leadership, and the patients and families affected by this disease, among many other stakeholders.

It is important to note that many investigators around the world have begun to address these challenges, with more than 600 interventional and RCTs currently registered in ClinicalTrials.gov related to COVID-19 and results expected to start emerging in the peer-reviewed literature imminently [8]. As an illustrative example of one such initiative, we have applied our translational science infrastructure to support of COVID-19 research at our institution. In times of less chaos, Vanderbilt University Medical Center invested significant effort into development and refinement of research infrastructure and capabilities as described above and now is positioned, like others, to help create an evidence base, instead of relying on (unsupported) belief. As a result, we have made the decision to not pursue open-label studies during this pandemic and instead, within 7 weeks (March 13 through May 1), we have 10 randomized, blinded, and controlled interventional clinical trials activated [9–18].

The global healthcare community, including clinicians, healthcare organizations, patients, government entities, and payers, requires *evidence* for understanding the utility of any therapy in the current pandemic. We, as translational scientists, are positioned and committed to support and conduct high-quality research, and we have the ability to assess these approaches with rigor and speed to ensure that helpful interventions move forward, and unhelpful or harmful interventions are discarded. Only in this way can we maintain public trust in research and impact the health of millions of people now and in the future, as new health challenges continue to emerge. Conducting research when feasible while treating those in need is an opportunity and obligation.
